# Building Career Development Skills for Researchers: A Qualitative Study Across Four African Countries

**DOI:** 10.5334/aogh.2759

**Published:** 2020-04-13

**Authors:** Halima Okewole, Christopher Merritt, Walter Mangezi, Victoria Mutiso, Helen E. Jack, Thalia C. Eley, Melanie Abas

**Affiliations:** 1 Barts and the London School of Medicine and Dentistry, London, UK; 2 Centre for Global Mental Health, Institute of Psychiatry, Psychology and Neuroscience, King’s College London, London, UK; 3 University of Zimbabwe College of Health Sciences, Avondale, Harare, ZW; 4 Africa Mental Health Foundation (AMHF), Nairobi, KE; 5 Department of Medicine, University of Washington, Seattle, US; 6 Social, Genetic and Developmental Psychiatry Centre, Institute of Psychiatry, Psychology and Neuroscience, King’s College London, London, UK

## Abstract

**Background:**

Career development skills are widely advocated as part of research capacity building and strengthening efforts. However, there is a gap in knowledge on their acceptability in low- and middle-income countries.

**Objective:**

This study aimed to examine how a group of 16 early-career researchers in sub-Saharan Africa experienced a career development skills course and how they perceived the utility of the course.

**Methods:**

Sixteen early-career researchers registered at universities in Ethiopia, Zimbabwe, Malawi, and South Africa took part in the year-long Academic Competencies Series (ACES) course. ACES comprised ten modules covering mentoring skills, work-life balance, career strategy, teamwork, presentation skills, teaching, academic writing, engaging policy makers, grant-writing, and digital media. ACES was delivered through face-to-face workshops and via webinar as part of a broader mental health research capacity-building programme. In-depth interviews following a topic guide were conducted with participants. Interviews were recorded and transcribed verbatim. Data were analysed using Thematic Analysis.

**Findings:**

All ACES participants were interviewed (9 male, 7 female). Participants were PhD students (14) and post-docs (2). The main themes identified throughout the course were 1) Growth, in both personal and professional life; 2) Application of training, often in innovative ways but with notable constraints and obstacles; and 3) Connection with colleagues, where researchers learnt from each other and from experts, building confidence in their new skills. Participants described how face-to-face contact enhanced the perceived quality of their learning experience. Barriers included logistical obstacles to applying training, such as limited resources and being at an early career stage.

**Conclusions:**

We found that research career development skills training was highly acceptable for early-career researchers in four African countries, and was perceived as having facilitated their personal and professional growth. Our findings suggest that courses like ACES can be applied successfully and innovatively in low-income settings.

## Background

Developing the skills of researchers is a vital aspect of capacity building and can help increase the quality and impact of research from low- and middle-income countries (LMICs) [1,2]. In addition to technical competencies such as systematic reviewing, study design and working with data, training in career development skills [3] such as communication, work-life balance, leadership, and mentoring is likely to be important in positioning researchers for success [4,5].

Several large-scale projects based in high-income countries (HICs) have used stakeholder working groups to develop models of desirable career development competencies [6,7,8]. Small-scale studies have demonstrated that training in career development skills builds competencies in specifically targeted areas such as scientific writing and presentation skills [9,10,11]. Other studies have tracked metrics such as grant income, publication, and presentation numbers using survey and feedback methods, indicating positive impacts of career development training, but without randomisation of participants or control groups [12,13,14]. A handful of qualitative studies and commentaries have also described psychological benefits of such training, including greater perceived support, more motivation, and stronger identity as a researcher [5,15,16,17]. However, there have been few rigorous studies of career development skills training.

A recent systematic review of 22 studies on researcher development interventions (both technical and non-technical) by Mazmanian and colleagues found that less than half of studies (40.9%) reported improvements in competence, including writing, presenting, analysing data, and research practice. The review also concluded that most studies offer weak evidence of cause and effect for benefits of training, and little information on how researchers apply such training interventions [18]. Moreover, all studies in the review were conducted in HICs, mostly in North America, Europe, and Australasia.

LMICs have a great need for research capacity building due to their high burden of disease, large treatment gap, paucity of existing research on epidemiology, and effective interventions, and limited numbers of trained researchers [2,19,20]. LMICs also experience significant barriers to conducting research and translating it into clinical practice, including lack of money, institutional weaknesses (such as lack of mentorship and absence of publication ‘culture’) and ‘brain-drain’ (out-migration of qualified and talented health professionals) [21,22,23]. Though research capacity building and strengthening is widely considered important in LMICs, a systematic meta-narrative review noted barriers to its implementation, including limited translation of research into practise and lack of a systems approach [24]. This review advocated for ‘soft skills’ or transferable skills training, i.e. career development competencies for researchers, such as leadership, teamwork, stakeholder engagement, and mentoring [24]. These skills gaps have been highlighted elsewhere specifically for sub-Saharan Africa (SSA) [25,26,27].

Despite this recognition of need for career development skills in LMICs, literature on such courses is limited, particularly on the effectiveness and acceptability of interventions. A review of 148 World Health Organisation (WHO) grantees in LMICs suggested that training in science writing and stakeholder communication produced an estimated 20–30% increase in self-rated competencies in these two domains, but gave no details of how training altered self-assessed competency [28]. Some capacity-building projects in SSA have included courses on career development skills, including leadership and management [29], communication [30], grant writing [31], science writing [32], and mentorship [33]. However, the majority of these papers focussed on technical research training and did not present any outcome data, with only one offering basic qualitative feedback on trainee experience. To our knowledge, no LMIC studies have allowed participants in career development skills training to reflect in depth on their experiences of the course and its application in their professional and personal lives following training.

## Objective

This study aims to 1) understand participant experiences in a career development training course, thereby giving a window into the acceptability of such training; and 2) investigate how researchers perceive applying career development skills training in their jobs. These findings will inform potential scale up of such courses across capacity building programmes in LMICs.

## Method

### Setting and Programme Description

The Institute of Psychiatry, Psychology, and Neuroscience at King’s College London (KCL) has, since 2008, run an award-winning career development research skills training course called THRIVE, for early- and mid-career researchers. THRIVE was set up as an annual opportunity with the aim being to position researchers for success in a competitive international research environment. It takes a cohort of 20 researchers through 8–10 workshops on a range of skills, each workshop lasting six hours. In 2016, MA and CM worked with THRIVE course leaders, including TE, to adapt THRIVE for co-delivery with African partners as part of KCL research capacity building activities.

The new course, called ACES: Academic Competencies Series, comprises a series of ten workshops run over a year. Topics include presentation skills, career strategy, digital communication, teamwork, work-life balance, teaching, engaging policy makers, mentoring, grant writing, and academic writing (see Appendix 1). Six topics are delivered through face-to-face, full-day workshops, and four online as 2-hour webinars. Merritt et al [34]. describes the adaptation, implementation, and course content in detail. ACES participants are paired up and asked to meet as peer-mentoring dyads every two months while the course is running, to reinforce skills taught through ACES and offer each other peer-mentoring around career issues.

ACES was offered to two cohorts of early-career researchers (ECRs) registered for PhDs or post-doctoral training at universities in Ethiopia, Zimbabwe, Malawi, and South Africa. Seven ECRs took part in the first cohort and nine in the second cohort. All researchers had been selected competitively for a fellowship with the African Mental Health Research Initiative (AMARI) [35]. AMARI aims to recruit, train, and support 50 postgraduate mental health ECRs at MPhil, PhD, and Post-doctoral levels across four countries: Ethiopia, Malawi, South Africa, and Zimbabwe. AMARI hopes to develop a critical mass of trained and networked ECRs who will form part of the next generation of health research leaders in SSA. AMARI provides its ECRs with tuition, a stipend, local and international supervisors, research funding, and additional training in research methods and grant writing.

### Design

A qualitative design with semi-structured interviews was chosen because researchers’ experiences in training courses and their perspectives on career impact may be nuanced and complex, best explored through the rich detail offered by qualitative interviews. Given the paucity of information on career development skills training in LMICs, qualitative interviews help develop hypotheses about how these skills are applied in research in resource-limited settings and what quantitatively measurable outcomes they may affect.

### Participants

All ECRs who had completed the ACES course at the time of the study were eligible to participate. Since there were only 16 ECRs (14 PhD, 2 post-doctoral), total population sampling was used. Ethical approval was sought and given by KCL Research Ethics Office. Once the senior faculty leading the AMARI programme in Ethiopia, Malawi, South Africa, and Zimbabwe had each approved the study, we contacted all 16 ECRs directly by email to inform them of the study and seek their consent to be interviewed. All gave written consent to participate.

### Data Collection

Interviews were conducted online using video-call platforms (either Skype, WebEx, or WhatsApp; participant was offered choice). Where participant internet bandwidth was limited, voice calls were used in two cases. The first author, who was based in the UK and not part of AMARI, conducted all interviews. The topic guide (see Appendix 2) for the semi-structured interviews was developed between all authors. This was informed by the WHO ESSENCE framework [36], Vitae’s participant evaluation template [7], the Five Levels of Professional Development Evaluation [37], and the Impact Framework [38]. For example, questions focused on participants’ perceptions of the benefits and limitations to training, any resultant change in attitude and behaviour, and use of new knowledge and skills. Overall, general perception of the course was solicited, followed by specific questions around each workshop. Prompt questions were developed and used to elicit detailed responses. Interviews were digitally recorded and transcribed by the first author. The second author sampled three transcripts randomly and read them while listening to the recordings to ensure transcription fidelity.

### Analysis

Thematic Analysis (TA) was chosen as the methodology to facilitate the identification of patterns across the data set rather than prioritising subjective experience, as in phenomenological methodologies [39]. Several authors in our team had extensive prior experience with qualitative research and supervised the analysis process and provided input on the code list. The authorship team brought their diverse backgrounds to the interpretation of qualitative data, including five different countries of origin (three in Africa) and experience across professional fields of medicine, psychology, and psychiatry.

The first and second authors read three of the 16 transcripts independently and noted down initial codes, descriptors that summarized verbatim quotes. They then met to compare initial codes. This process was repeated with a further three transcripts until a consensus code list was developed using a constant comparative approach [40]. This list was shared with the wider authorship team. The first author then coded the remaining transcripts using the consensus code list. Following this, the first and second authors met to discuss themes and sub-themes identified in the coding process, meeting again to refine themes and ensure they had adequate ‘uniqueness’ to remain separate rather than be merged. Finally, theme names were developed to capture the essence of themes and sub-themes. Several verbatim quotes were extracted from transcripts to illustrate each sub-theme. NVivo software [41] was used to manage the transcript data, codes, and illustrative quotes.

## Results

As shown in Table 1, more participants were male, the majority came from Ethiopia and Zimbabwe, and nearly all were doctoral students. Workshop attendance ranged from 75–100% of those invited to each session (mean = 90.2%; see Merritt et al.) [34], indicating good accessibility for the training. Interview length ranged from 23–49 minutes.

**Table 1:** Participant characteristics.

		N	%

Gender	Female	7	43.7
	Male	9	56.3
Country	Ethiopia	6	37.5
	Malawi	3	18.8
	South Africa	1	6.2
	Zimbabwe	6	37.5
Research level	PhD	14	87.5
	Post-Doctoral	2	12.5
Professional background	Medical (doctors, nurses)	5	31.3
	Clinical Psychology	3	18.7
	Other Health Professional*	3	18.7
	Academic	3	18.7
	Public Health	2	12.5

* ‘Other Health Professional’ includes occupational therapy (n = 1), physiotherapy (n = 1), and clinical officer (n = 1).

Three themes were identified from the data: 1) Growth: participants reported both professional and personal growth as a result of the training; 2) Application: participants reported using learned skills in their daily work as well as barriers to doing so; and 3) Connection with colleagues: participants emphasised the importance of physical co-location with each other and the module facilitators. Each theme comprised two sub-themes, shown in Table 2. Supplementary quotes are presented for each theme in Appendix 3.

**Table 2:** Study themes and sub-themes with associated codes.

Theme	Sub-theme	Code

Growth	Professional	New theory
		New skills
		Long-term development
	Personal	Self-reflection and insight
		Self-confidence
Application	Innovation	Direct
		Indirect
	Constraint	Obstacles
		Future intention
Connection	Co-location	Face-to-face
		Practice builds confidence
	Mutual benefit	Learning from others
		Using skills with others

### 1. Growth

This theme captures participants’ experiences of professional and personal growth as a result of the course. Professionally, participants reported the acquisition of new theory and skills relevant to their research careers, often citing specific ACES sessions such as mentoring for career development:

…mentoring is like a career development kind of thing so, and it’s mutual […] I am mentoring someone, I am benefiting, and the mentee is also benefiting (Participant 9, hereafter P9).

Communication skills were also cited as a key area for professional growth by many participants, in areas such as presentations and digital media use:

I saw […] lots of dramatic changes in many of the fellows […] after the presentation skills course (P9).

I didn’t realise that I can actually use social media to also advance my capability […] in terms of communicating my research findings (P7).

These new ideas and skills were described as being applicable not just for a PhD, but also longer term across the span of a research career:

It’s really skills […] which you will always have for the rest of your life (P3).

These are the […] tools you need, like the tools of the trade (P6).

Additionally, as a result of these skills, participants often described personal growth from boosted self-confidence after the workshops, both generally and in specific skill domains after training:

…before I started the ACES, I was just a novice […] but now I can actually stand up and give my presentation in a confident way (P4).

I’m so confident that I can submit [a paper] by the end of the year. It’s because of the writing workshops (P5).

Participants also described a more personal sense of growth, stemming from processes of self-reflection and insight which arose from several workshops:

…all the ACES courses that we did are applicable to me as a researcher and a lecturer, and some of them are applicable to me as a human because they were more like life lessons (P2).

One fellow highlighted this personal growth through developing awareness of their own strengths after the teamwork session:

I was more aware about what I’m really good at, what I should be doing […] what I should concentrate on. Not just in my personal life, but even career-wise (P3).

Others spoke about the impact of the work-life balance session in making them reflect and re-evaluate their lifestyle and priorities in the context of a research career:

Since [the workshop] I have considered that I […] need to have also life beyond the dissertation (P12).

For other participants, the sense of growth as a result of some workshops was more limited. Adequate workshop time and practical exercises were mentioned as key ways to embed learning and build confidence; where these were reduced, growth was perceived as lower:

…the information was so much and […] within the space of time that it was allocated, it was difficult to grasp some of the concepts (P15).

…still I cannot say confidently I can write a good grant proposal (P11).

### 2. Application

This theme captures the ways that fellows were able to apply their new ideas and skills in daily work and barriers to doing so. Almost all participants reported significant changes in their working practices as the result of their training, directly applying new skills from across the ACES course:

I’m using that presentation skill, preparing a PowerPoint skill for the seminars, presentations at some different scientific conferences (P10).

…now we [participant and mentor] are working on my paper […] you assist each other and we’ve also been just being there for each other even for personal issues, academic issues (P5).

They also described applying a new idea or technique indirectly, beyond the original scope of the workshop. This was particularly the case with mentoring, where fellows applied their training in other professional and personal spheres:

I’m mentoring my daughter, I’m mentoring a friend of mine, so it’s […] working out well (P4).

…I’m transferring the [mentoring] skills to working with students and working with my colleagues (P9).

Participants spontaneously reported innovative uses for learned skills such as the teaching and presentation skills in other domains of their professional lives outside of research:

…when we’re training field workers or counsellors then they [teaching skills] come in handy (P1).

…[presentation skills] is helping me in doing my day-to-day activities when I’m teaching student lectures (P10).

Alongside many examples of successful innovation, participants also spoke about constraints in applying their new skills and ideas. These obstacles took a variety of forms: time constraints, lack of opportunity, limited resources in their setting, being too early in their PhD programmes, and insufficient confidence.

We don’t necessarily have […] the facilities… and […] factoring [in] the number of students, it may not be necessarily feasible to actually do small-group teaching (P7).

…but still we are not [yet] producing papers from the PhD programme so… I’m not using it (P8).

As the previous quote illustrates, despite these barriers all participants voiced the intention to use these skills in the future, particularly at an appropriate point in their fellowships or later careers:

…we’re planning now to find ways to engage policy makers […] I think we’ll do something for the future (P9).

…next time when I want to write a grant, I need to make sure that I research thoroughly, on the funders, on the project that I will be applying for (P5).

### 3. Connection with colleagues

The third theme captures the importance of interaction among participants and between participants and session facilitators for learning and support. Co-location was crucial to this connection, with participants frequently commenting that the deepest learning came from face-to-face workshops:

Maybe the face-to-face ACES that we had were much more helpful and more interesting and more interactive than the webinar […] and we could actually discuss things among ourselves (P4).

Though webinars were interactive, they were not perceived as being as ‘connected’ as a physical workshop:

…the challenge now comes in where the lecture is […] there in UK and you’re here [Africa] and I think maybe some of the concepts would have made sense […] more sense if you’re there face-to-face (P15).

…some of the webinars, we have difficulty in participating all the way because of the network here, because the network is interrupt[ed] very much (P11).

The connection that co-location provided generally helped fellows build confidence through practising skills together:

…the session [was] a chance to rehearse and present our own work […] we developed our own presentations […] and then we got feedback […] you’re more confident because now you can handle questions (P16).

When sessions were shorter or not conducted in person, some participants reported issues:

I definitely need more practise … [it] would actually require maybe […] a five-day workshop […] where people would actually practice the actual [grant] writing together (P7).

Participants specifically noted the importance of working with more experienced professionals, establishing connections that helped find novel ways to approach their work and left them feeling inspired:

…you identify better when you see somebody from your professional background who has done it and can hold your hand, can understand the nitty-gritties of your field (P2).

…learning from others who’ve done it before […] these are like incredible people who do incredible work […] it’s quite amazing (P6).

In addition to this, many participants reported using skills acquired through ACES to make connections with others in their wider professional communities, including other researchers and stakeholders:

…I’ve also even developed a network with other people who saw me being active [on Twitter] (P2).

…we invited her [the policymaker] for the meeting so that we could find out from her […] what the ministry is targeting and […] how we can include the ministry focus in our endeavours (P5).

Similar to these examples, most participants reported having broader professional networks as a result of applying learning from the ACES training course.

## Discussion

This study’s aim was to understand the perspectives of ECRs from four African countries on participation in a training programme for career development skills. The main findings were that researchers almost universally found the new skills useful and perceived that they could apply them broadly in their professional and personal lives, which increased their confidence and perceived professional success. However, their application of the new skills was primarily limited by resource constraints. To our knowledge, this is the first study that used semi-structured interviews to investigate a career development research skills training programme in any LMIC. The findings may inform the development and implementation of future capacity building of this type.

Previous small-scale studies in HICs have illustrated the perceived utility of learning career development skills that comprise ACES [5,10,11,14,16,17,42,43]. For example, Angelique et al. (2002) reported the development of collegiate connections through peer mentoring [15]. Additionally, focused and regular mentorship has helped ECRs maintain good work-life balance, stimulate projects, and increase likelihood of grant success [5]. In the present study, participants highlighted these and other benefits of peer mentoring. For example, some participants reported that deadline-setting with their mentor motivated them to complete assignments.

Although literature on career development skills training in LMICs is limited, two studies featuring qualitative feedback matched our findings. Mangezi et al. (2014) introduced research mentorship for psychiatrists in Zimbabwe [33]. They found that in-country teaching and mentoring were relevant to trainees; teaching methods need to be feasible given resource constraints; and that mentorship fostered leadership in education, a finding which was replicated in the present study. In the present study, opportunities to interact with each other and the facilitator in face-to-face sessions were perceived by the ECRs as playing a key role in the benefits which they felt they had accrued through taking the ACES course. Despite the increase in resources needed, this theme highlights the importance of co-location, where possible, to ensure extra learning occurs and greater benefits are accrued. Nakanjako et al. (2015) described leadership training in Uganda, including modules on communication, grant writing, leadership, and structured mentorship [30]. Participants reported professional growth as they used the skills acquired to take on new responsibilities and to innovate in their research practice post-leadership training, supporting our themes of growth and application respectively.

This study has several novel findings that can inform training for ECRs. Firstly, while professional growth is often cited in studies of capacity building, personal growth has rarely been reported. We found that fellows described innovative applications of skills in new domains. For instance, mentoring skills were not just confined to their professional roles but were reported as useful in their personal lives to mentor relatives and friends. Additionally, many were utilising presentation skills not just for research dissemination but also in teaching. Dugani et al. (2018) reported the high prevalence of burnout among various healthcare professionals in LMICs [44]. ACES modules such as work-life balance and career strategy could help combat burnout. These findings highlight the benefits of training programmes like ACES, with scope for creativity in application of the skills beyond their original intended function.

Secondly, we identified three key barriers to applying skills from the ACES programme including a lack of resources, internet connection issues, and fellows being at early stages of their research careers. Resource barriers were especially pronounced in the teaching skills course and use of digital media. Limited multimedia facilities for teaching as well as large class sizes reduced ECRs’ capacity to engage in small group teaching, while restricted internet access and government internet blackouts limited social media engagement.

In summary, we adapted a course designed for early- to mid-career researchers in the UK and applied it to ECRs in four African countries. The ‘growth’ theme highlighted the acquisition of new professional skills, indicating the lack of opportunity many of these ECRs encountered before joining their doctoral programs. This study also shows that some skills, such as academic writing and grant writing, were introduced at too early a stage, when researchers were not ready to publish or apply for grants (see ‘Application’ theme). Future career development training programmes should take into account these barriers, and match interventions to researchers’ career stage.

## Limitations

This was a relatively small qualitative study (N = 16), so may be of limited generalisability, but census sampling was used and theoretical saturation was reached. There was only one coder (HO) for ten of 16 transcripts, but the first six transcripts were discussed extensively with collaborators and experienced qualitative researchers (HJ, CM, MA) to agree a coding frame.

Whilst more of the participants were male, there was still reasonable representation of females (44%), and this higher proportion of males reflects the gender differential among researchers in many countries. The themes across the whole study may mask subtle differences between genders, project countries, or institutions although these were not explicitly mentioned during interviews. However, such comparisons are beyond the scope of a small qualitative study and may have raised confidentiality issues around identifiability of participants. A larger quantitative study would be more appropriate to investigate such differences.

Finally, participants may have felt pressure to respond positively due to social desirability and acquiescence bias when giving feedback on training. However, this was mitigated by interviews being conducted by someone outside ACES (HO). Also, assurances of anonymity were provided; the freedom with which participants shared views on more negative experiences (i.e. barriers) suggests this was not a significant source of bias.

## Future Directions

Awareness of challenges faced by participants in this study could highlight areas for improvement when designing future training programmes. Ways of addressing these challenges might include delivering more sessions face-to-face, therefore avoiding internet connection issues, ensuring courses are appropriate to the LMIC setting in terms of resource availability, and tailoring sessions to the career stage of fellows.

Longer-term follow up within AMARI for quantitative measures of impact such as publications, presentations, grant applications, courses taught, and stakeholder engagement is needed. It is important to explore whether the personal experience of ACES matches the implementation of its skills long-term. Furthermore, it is imperative to test different educational methods within a course like this to find the ideal balance between practical and didactic teaching and between in-person and online delivery methods and evaluate these against key outcomes such as publication and successful grants. Beyond this, future studies should evaluate outcomes like work-life balance and burnout as well as evaluating the cost-effectiveness of career development skills training.

## Conclusions

The present study illustrates the perceived benefits of including career development skills training within a research capacity-building project. Participants found the ACES program useful and applicable across their professional and personal lives. Additionally, they noted the importance of face-to-face teaching as a key method of learning. Our results could inform future capacity-building projects for research training in LMICs, where career development skills are needed.

## Data Accessibility Statement

Original interview recordings are not available as per confidentiality and ethical requirements. Anonymised qualitative transcripts are available on request. Non-copyrighted ACES materials such as workshop lecture slides are available from the corresponding author on reasonable request.

## Additional Files

The additional files for this article can be found as follows:

10.5334/aogh.2759.s1 Appendix 1. AMARI ACES workshops summary.

10.5334/aogh.2759.s2 Appendix 2. AMARI ACES interview guide – Research fellows.

10.5334/aogh.2759.s3 Appendix 3. Supporting quotes for themes.

## Figures and Tables

**Table:** 

ACES:	Academic Competencies Series
AMARI:	African Mental Health Research Initiative
DELTAS:	Developing Excellence in Leadership, Training and Science
HIC:	high-income countries
KCL:	King’s College London
LMIC:	low and middle-income countries
SSA:	sub-Saharan Africa
WHO:	World Health Organization

## Author Information

All authors confirm that they had access to the data from this study, and that they each had a role in writing the manuscript.
