# Supplementary material for: Building Career Development Skills for Researchers: A Qualitative Study Across Four African Countries
Source: Ann Glob Health. 2020 Apr 13;86(1):40. doi: 10.5334/aogh.2759 (PMC7164382; doi:10.5334/aogh.2759)
Supplement: Appendix 1. — AMARI ACES workshops summary. [file agh-86-1-2759-s1.pdf]

Appendix 1: AMARI ACES workshops summary

SELF-DEVELOPMENT

- **Mentoring**: Fellows learn the skills and techniques to be an effective mentor before entering into a year-long peer mentoring scheme with another AMARI fellow. They also learn how to be mentored by others, i.e. how to be a mentee. The course is practical and experiential, with fellows having the chance to engage in mentoring conversations during the day and begin to identify personal goals.

- **Work-life balance**: Activity management techniques are discussed along with testimonies, stories and Q&A with experienced African researchers who have coped with the challenges of finding balance. New ideas for working efficiently as well as looking after yourself are covered. Many fellows report that this is one of the most innovative and useful sessions on the course.

- **Teamwork**: After a general introduction to teamwork theory, fellows take personality assessments with a trained psychologist (either the Big Five or Myers-Briggs assessments), then look at how their own individual personalities influence team performance. We also cover conflict management, using the Thomas-Kilmann questionnaires, before discussing different approaches to resolving conflict. There is the option to engage in role playing difficult situations with colleagues to practise conflict management.

- **Career strategy**: We begin by setting out the elements that are needed for success in research careers. Fellows learn how to develop their research identity, set strategic goals for themselves, then how to build and mobilise networks to reach those long-term aims. This module was developed by international expert on research and academic careers Prof Shelda Debowski.

ENGAGEMENT

- **Presentation skills**: This workshop focusses on learning how to give effective verbal presentations, including pitching proposals and describing research work, and how to create strong visuals to support talks. The session is experiential and can include practising oral presentations, and hands-on tuition for PowerPoint skills.

- **Digital media**: This session shows fellows how to use digital platforms to disseminate their work, build their profiles, and engage safely in online debate through social media, blogs and open science. A session on briefing the media is included, covering approaches to communicating science to non-specialist audiences, with experiential exercises.

- **Engaging policy makers**: This workshop discusses how best to translate research findings into practice by working with policy makers to influence and guide their decision-making on health service delivery. We draw on examples from Africa and wider LMIC settings to demonstrate the process. Fellows engage in an extended exercise to develop and pitch a proposal to policymakers.

- Teaching: fellows learn the theory of teaching adults, and how to tailor lectures and classes to a range of audiences, including working online and in low-resource settings. The workshop draws on experiential learning to give fellows the opportunity to develop and deliver sessions using a range of teaching techniques.

WRITING

- Academic writing: this five-day residential and experiential workshop teaches the theory of effective written communication, before giving dedicated time to fellows to write up part of their research for publication. They receive individual facilitator input and peer feedback. The academic writing course is led by Dr Helen Jack of the University of Washington.

- Grant writing: fellows learn the basics of developing a grant proposal and how to 'think like a reviewer' when writing and submitting an application. Our workshop focuses on smaller grants for early-career researchers and draws on case studies to illustrate the principles. This session can be delivered as a shorter, more didactic component, or a longer, more experiential workshop with fellows bringing and working on their own proposals.
