# Supplementary material for: Building Career Development Skills for Researchers: A Qualitative Study Across Four African Countries
Source: Ann Glob Health. 2020 Apr 13;86(1):40. doi: 10.5334/aogh.2759 (PMC7164382; doi:10.5334/aogh.2759)
Supplement: Appendix 2. — AMARI ACES interview guide – Research fellows. [file agh-86-1-2759-s2.pdf]

## Appendix 2: AMARI ACES interview guide – Research fellows

<u>General</u>

- What were the most useful sessions for you throughout the ACES program?
- Give examples of where you've used skills learnt from these sessions?
- What (if any) courses do you feel are missing from the course schedule that are important to you becoming a competent and confident researcher?
- What (if any) of the courses do you feel should be removed from the course schedule?
- What do you remember about the quality of presentations?

*Prompt: Number of slides*

*Opportunity to ask questions and engage with other fellows*

- To what extent do you feel these sessions are applicable to your career as a researcher?
- With regards to your confidence before these courses, how empowered do you feel after having attended all these sessions?

<u>Mentoring</u>

- What do you remember from the mentoring training session?

*Prompt: Length; Information provided; What you took away?*

- How useful do you think it was to have had the training?

*Prompt: Any examples of where you've used a skill you learned from the training?*

<u>Presentation skills</u>

- How have your presentation skills changed since you've had the session?

*Prompt: What skills are you using in your presentations now that you learnt from the session?*

*What feedback have you had on your presentations since the session?*

<u>Use of Digital Media</u>

- How has your use of digital media (blogs, twitter, Facebook, popular press etc.) changed since the session?

*Prompt: What benefits / disadvantages are you accruing from using Digital media?*

- Have you made any plans to use it for future work?

Teamwork

- What did you learn about yourself from taking the Myers-Briggs test?
- What did you take away from the presentation on teamwork?
- Have you used any skills you learnt from the session to address any challenges since?
    - If yes, Example?

Work-Life Balance

- What changes have you made to balance work and life since the session?

*Prompt*: To what extent do you feel you now have a better-work life balance?

*Any plans to make changes to you haven't yet made?*

Grant writing

- Had you ever done a grant application prior to the session?

*Prompt: Can you comment on any success or otherwise?*

- Have you applied for any grants since the session?

*Prompt: How prepared did you feel after having done the session?*

Teaching skills

- Do you currently do any teaching?

*Prompt: What teaching do you do currently?*

- How has your teaching style changed since the session?

*Prompt: Examples? Any feedback from your students?*

Policy Making and Health Systems

- Were you engaging policy makers prior to these sessions?
- Did you learn to do anything differently from this session?
- Are you currently engaging with policy makers?

*Prompt: If yes, what skills are you utilizing?*

Writing workshop

- How confident do you feel per your academic writing skills since you've had the session?
- In what way has the workshop affected your publishing track record?
