# Supplementary material for: Building Career Development Skills for Researchers: A Qualitative Study Across Four African Countries
Source: Ann Glob Health. 2020 Apr 13;86(1):40. doi: 10.5334/aogh.2759 (PMC7164382; doi:10.5334/aogh.2759)
Supplement: Appendix 3. — Supporting quotes for themes. [file agh-86-1-2759-s3.pdf]

Appendix 3: Supporting quotes for themes

Theme	Sub-theme	Supporting quote [specific code]
Growth	Professional	…teaching skills […] grant-writing and […] working as a team […] these are some of the courses that […] I was able to use […] immediately (P16) [New skills]<br><br>I didn’t know much about mentoring before that […] I was not sure if it is effective or not, but [now] I think that helps us a lot (P11) [New theory]<br><br>[…] to me it was a eureka [moment] in terms of learning […] I think it has made me realise that beyond the publications, beyond the good research, I also need to be able to disseminate these […] findings to the wider public (P7) [New theory]<br><br>…mentorship, the work-life balance sessions […] were also very impactful […] because they live beyond the PhD (P2) [Long-term development]

	Personal	…it was insightful because I think with academic life at times it's easy to get lost with your studies, with your publications and all and neglect […] your immediate family at times (P7) [Self-reflection and insight]<br><br>I think when you […] really prepare especially for things like presentations, it makes everything […] easy, you're more confident because now you can handle questions that people [are] asking […] I'm progressing very well, I think I'm more confident than before ACES (P16) [Self-confidence]<br><br>…the ACES program […] generally improved my skills, […] my competencies and my confidence (P9) [Self-confidence]
Application	Innovation	…there are two papers I'm working on and I'm benefiting […] utilising skills from the writing workshop (P2) [Direct]<br><br>I was really Twitter-shy. And after this [Digital media] session […] I'm now active on Twitter, I tweet, I re-tweet stuff that's relevant to my work in terms of mental health (P6) [Direct]

		I'm using some of those [mentoring] concepts when I'm supervising Master's students (P1) [Indirect]<br><br>I can say about the teamwork, because I also sit in […] three boards for [healthcare delivery] in [country], I started to realise that we run into problems when we don't use people's characteristics [for] portfolios where they are best suited (P13) [Indirect]
	Constraint	Like for example, with the policy writing, I think, yeah I'm…I wouldn't say I'm too confident in that erm, yeah because I think the time allocated was just too short (P3) [Obstacles]<br><br>I've been trying to use […] both Facebook and Twitter […] I have not involved [them] as much […] because of the internet problems here, but I'll use them in the future, I'm planning to use them (P12) [Obstacles / Future intention]<br><br>I've started the introduction […] when I'm done with data collection, I'll be able to put in the whole paper together (P4) [Future intention]

		…we're planning now to find ways to engage policy makers. So, I think we didn't do much […] so far but I think we'll do something for the future (P9) [Future intention]
Connection	Co-location	…these are sessions that are very interactive in nature, they're not didactic […] we talk, we discuss, we get input […] [we] also get to react and make our contributions […] it's quite interesting (P6) [Face to face]<br><br>…the [sessions] we had face-to-face […] they were more […] interactive and we could actually discuss things amongst ourselves as well as with [the facilitator] (P14) [Face to face]<br><br>I think I'm much more confident now because during the workshop, we would […] read […] particular sections of a paper or a particular paragraph from somewhere and […] have examples of what's good writing and what's bad writing […] [and] how to […] use good grammar […] by doing that and really going back to those basics, now when I write […] I could really critique what I was writing and then apply all that we were learning (P3) [Practice builds confidence]

	Mutual benefit	…when [it] is presented with an experiential type of charge to it, I think I remember […] the presentation, mentorship done by [facilitator], you could relate to his own work and his own life experience, which […] gave it life […] even the work-life balance they had […] renowned researchers in the field, people whom we could identify with and that was what made it very much of good quality (P2) [Learning from others]<br><br>… I've got one supervisor who doesn't live in the country as well, so, getting a peer-mentor has been very helpful in that at least I get someone to talk to, our research [is] quite similar, […] So, I've found that very, very helpful because I've got someone to bounce my thoughts on (P5) [Learning from others]<br><br>I've been involved with writing papers with other people on some of the other projects I've worked on […] and […] I'm trying to guide a couple of my students to publish now (P1) [Using skills with others]<br><br>I'm using some of the [teamwork] skills in working with friends […] sharing tasks, allocating tasks and […] discussion on the points back and reaching an agreement (P12) [Using skills with others]
